# The Working Girls

**Published:** 1911-11

**Authors:** 


### EDITORIAL DEPARTMENT.

### THE WORKING GIRLS.

A NOBLE EXAMPLE WORTHY OF IMITATION.

I dare say few of my readers have given a thought to or reflection on the condition, environment and necessities of the hundreds,—yea, thousands of young girls in the towns and cities who, thrown upon their own resources, orphans, perhaps, have to earn their own living. Their condition should appeal to every parent, to every man with a heart in his bosom and worthy the name of man. In the stores and shops, the laundry, the bindery, the telephone exchanges, there are hundreds of girls, some of very tender age, who are bravely battling with life, striving to maintain their purity and self-respect upon $3 to $5 a week! They must be lodged, must eat and dress—yes, they must present a neat appearance to wait upon the ladies or lose their place—on 50 cents a day. Suppose one should get sick, or lose her employment—what would become of her? Those who have homes, however humble, and parents, or perhaps a widowed mother, fare a little better; they have a shelter, at least. But many go from the country and, being unskilled, unqualified to teach or sew or do any skilled or special work—fill the lowest positions, and in some of the stores and shops are paid as low as 50 cents a day. They occupy a cot in the hall or stairway, or a cuddyhole, with some poor family in squalid surroundings; live on rice and molasses, cheap breakfast foods and canned goods, half starved, and they must launder their poor, scant clothes, perhaps after a hard day's work on their feet. They have no amusement, no recreation, go nowhere, have no time to read, have no books, and should a male friend wish to visit them, they have nowhere to receive him—they must go to the parks, the picture shows or on the streets. The vultures of society take advantage of their necessities and temptations, and take them to places of amusement, to the restaurants,—persuade them to take wine, perhaps, and "lend" them more money than they can make in a month by the hardest work. Do you wonder that they sometimes fall—that they are driven by hunger and temptation to lives of shame, to feed the houses more voracious and dreadful than Moloch, the fire god of the Ammonites? "Like the snowfall on the river," they are "white a moment, then gone forever." What follows every doctor knows too well. They can never escape, they are slaves for the remainder of their short and wretched lives. They contract the loathsome diseases, and dissolute husbands carry it into hundreds of homes; and infected wives, with wrecked health, bring forth blind or feeble children and go, sooner or later, to the gynecologist's table. Morrow says seventy per cent of the ovariotomies are for infective salpyngitis, and that ninety per cent of all adult males have venereal disease at some period of life.

The good women of Austin are working to save these innocents. Two years ago they had "tag day" and realized nearly three thousand dollars. Since then they have raised as much more, and have established a Girls' Co-operative Home. They purchased a fine property for $12,000, in the heart of the city, and have paid half of the purchase money.

In the home the girls are lodged in clean rooms and beds. Their earnings are clubbed and they are fairly fed. They are under the eye of a matron. They have a piano, books, magazines and papers, and when a friend calls they receive and entertain him in a respectable manner. Thus is their self-respect fostered, and thus are they enabled to rest, have recreation and be fitted for the labors of the morrow.

Thus aided and protected, they will in time become virtuous wives and mothers. It is so much better and easier to save a girl than to "rescue" her; to form a character than to "reform" it. Yet millions are spent by philanthropists for the foundation and support of "rescue" homes, but not a dollar for a saving home. To buy and endow this home would be a monument for some humanitarian more enduring than brass or marble. The altruists are not all dead, but those who would, can not; and those who can should be glad to aid in this great humanitarian work, the greatest of the age.

It is truly remarkable that a civilized people will ignore causes and continue forever to strive to remedy or cure evil results of causes easily preventable. We let our children burn their fingers then put salve on them. We pollute our streams with human excrement, then filter the water, and when typhoid fever breaks out, wonder where it came from. We permit flies to breed and put up screens to keep them out. We build palaces for the ever increasing insane, and strong prisons for the criminal, but permit the sale of poisons, liquor, morphine, cocaine and chloral, liquor being responsible for forty-six per cent of the former, and fifty per cent or more of crime,—and "Jones, he pays the freight."

That is, the taxpayers pay one million a year for the care of the insane in Texas, and God only knows how much for the criminal!

